# Effect of Universal Masking on Non–Severe Acute Respiratory Syndrome Coronavirus 2 Healthcare-Associated Respiratory Viral Infections

**DOI:** 10.1093/ofid/ofae617

**Published:** 2024-10-14

**Authors:** Satish Munigala, Patrick R Ching, Helen Wood, R J Waken, Josephine Fox, Heather Gasama, Robert Russell, Melanie L Yarbrough, David K Warren

**Affiliations:** Division of Infectious Diseases, Department of Medicine, Washington University School of Medicine, St Louis, Missouri, USA; Division of Infectious Diseases, Department of Medicine, Washington University School of Medicine, St Louis, Missouri, USA; Department of Hospital Epidemiology and Infection Prevention, Barnes-Jewish Hospital, St Louis, Missouri, USA; Division of Biostatistics, Washington University School of Medicine, St Louis, Missouri, USA; Department of Hospital Epidemiology and Infection Prevention, Barnes-Jewish Hospital, St Louis, Missouri, USA; Department of Hospital Epidemiology and Infection Prevention, Barnes-Jewish Hospital, St Louis, Missouri, USA; Department of Hospital Epidemiology and Infection Prevention, Barnes-Jewish Hospital, St Louis, Missouri, USA; Department of Pathology and Immunology, Washington University School of Medicine, St Louis, Missouri, USA; Division of Infectious Diseases, Department of Medicine, Washington University School of Medicine, St Louis, Missouri, USA

**Keywords:** COVID-19 pandemic, healthcare-associated respiratory viral infections, healthcare setting, medical face mask, respiratory viral infections

## Abstract

**Background:**

Respiratory viral infections are common and are a major cause of morbidity and mortality. We evaluated the impact of universal masking implemented during the coronavirus disease 2019 (COVID-19) pandemic on other healthcare-associated respiratory viral infections (HA-RIs) in an academic medical center.

**Methods:**

A retrospective cohort study was performed among all inpatients aged ≥18 years admitted between 1 May 2019 and 30 June 2022. Universal masking was implemented in May 2020 at our hospital and state-level mask mandates had been lifted by May 2021. We evaluated and compared the HA-RI rates, adjusted for monthly community-onset viral infections, during the premasking period, universal masking period, and post–community mandate period.

**Results:**

We identified 3015 patients (median age, 58 years; 48.0% males) with a positive respiratory viral test within 14 days prior to, or during, their hospitalization; 441 (14.6%) patients had an HA-RI. Rhinovirus/enterovirus (51.0%), parainfluenza virus (14.3%), coronaviruses (229E, OC43, HKU1, and NL63; 13.2%) and influenza (10.0%) were the predominant HA-RI viruses detected. The monthly HA-RI rate decreased 34.9% (95% confidence interval, 8.8%–51.8%) after the implementation of universal masking (0.71 premasking period vs 0.19 universal masking period vs 0.35 infections per 1000 patient-days in the post–community mandate period) while accounting for a drop in the community-onset respiratory viral infections using a structural time-series model analysis (*P* < .001), with no significant change in HA-RI rates with the relaxation of community masking mandate.

**Conclusions:**

Implementation of universal masking at our hospital was associated with a significantly reduced incidence of HA-RIs.

## Teaser

Implementation of universal masking at our hospital during the COVID-19 pandemic was associated with a significant reduction in the incidence of non-SARS CoV-2, hospital-associated respiratory infections, after adjusting for the prevalence of those viruses in the community.

Respiratory viral infections are common. In 2019, an estimated 17.2 billion cases of upper respiratory infection occurred globally [1]. Respiratory viruses can be transmitted in the hospital and are a major cause of morbidity. In the United States (US), an estimated 18 955 cases of healthcare-associated respiratory viral infections occur yearly [2]. These infections lead to increased length of hospitalization, unnecessary diagnostic studies and antibiotic use, increase in hospitalization cost, and mortality [3].

Hospitals employ multiple infection prevention strategies to prevent respiratory virus transmission. These include patient and staff screening, isolation and cohorting, cleaning and disinfection, hand hygiene, and personal protective equipment. Prior to the emergence of severe acute respiratory syndrome coronavirus 2 (SARS-CoV-2), universal masking of all healthcare workers (HCWs) and visitors had been examined in limited healthcare settings. In 2 hematopoietic stem cell transplant (HSCT) units, the incidence of respiratory viral infection significantly decreased during the universal masking period, compared to the premasking period [4, 5]. In the coronavirus disease 2019 (COVID-19) pandemic era, universal masking in healthcare facilities was widely implemented in the US. Several studies reported significant decreases in COVID-19 transmission among HCWs with universal mask use [6, 7].

The pre- and postpandemic studies had limitations. In studies performed prior to the COVID-19 pandemic, masking was implemented only in certain hospital areas, specifically HSCT units. In the COVID-19 era, even though masking was throughout the entire hospital, the focus of these studies was limited to the effect on SARS-CoV-2 transmission. Transmission of other healthcare-associated respiratory viral infections has not been as well studied. Although a healthcare network in Hong Kong showed zero rates of nosocomial-acquired influenza A, influenza B, and respiratory syncytial virus (RSV) infection in early 2020 after universal masking [8], this was only over a 3-month time period at the start of the pandemic and in the setting of extensive community control measures. The goal of our study is to determine the effect of universal masking on healthcare-associated infections due to respiratory viral infections (HA-RIs) other than SARS-CoV-2 over a prolonged time period that included lessening of COVID-19 mitigation measures in the community.

## METHODS

We conducted a retrospective cohort study of all adults 18 years and older hospitalized at Barnes-Jewish Hospital (BJH), a 1250-bed tertiary care academic medical center, who had a positive result from respiratory viral testing performed within 14 days prior to hospital admission and/or during their stay, from 1 May 2019 to 30 June 2022. Data on patient demographics, hospital encounter, and location where the testing was done were extracted from the hospital medical informatics database.

### Study Definitions

Healthcare-associated respiratory viral infections were defined as first positive test for a respiratory virus on day 3 or later of hospitalization.

Community-onset respiratory viral infections (CO-RIs) among hospitalized patients were defined as having a positive test for a viral respiratory pathogen either prior to day 3 of the index hospital admission or within the last 14 days prior to the index hospital admission in the outpatient or emergency department setting.

#### Intervention

Prior to the COVID-19 pandemic, the practice in the hospital was to follow Centers for Disease Control and Prevention transmission-based guidelines, which included hospital staff wearing an isolation mask when caring for patients with known or suspected respiratory viral infections [9]. During influenza season, visitors were advised via signs posted at hospital entrances to avoid visiting patients if they had a fever and/or respiratory symptoms. Visitors were not required to wear isolation masks. In response to the COVID-19 pandemic, universal masking was implemented at BJH on 1 May 2020. All individuals (ie, patients, visitors, contractors, and hospital staff) >2 years of age were required to wear an isolation mask in all hospital areas, including clinical and common areas (eg, main hallways, waiting rooms). Inpatients were required to wear a mask outside of their room and during transport, if medically able to tolerate it, and were encouraged to wear masks in their room when HCWs entered the room, whenever possible.

Community COVID-19 pandemic interventions in the St Louis region were similar to other regions of the United States and included closing schools and restricting public gathering in March 2020, with public health stay-at-home orders in place from late March 2020 until mid-May 2020. A community mask mandate was in effect from July 2020 until May 2021, with the relaxation of the masking mandate in June 2021 [10].

#### Outcome

The primary outcome was the incidence of HA-RI among adult inpatients per 1000 patient-days. As COVID-19 infections were predominant only during the post–universal masking period, the impact of universal masking on COVID-19 hospital-onset infections was not assessed and was excluded from the analysis. To account for potential confounding by overall community incidence of various respiratory viruses in the community, monthly respiratory virus community prevalence was calculated by the prevalence of CO-RI among patients admitted to BJH during the study period.

### Respiratory Viral Assay

Multiple respiratory viral assays were in use during the study period (Supplementary Table 1). Respiratory viruses that were analyzed for this study included rhinovirus/enterovirus, influenza (A, A/H1, A/H3, A/H1N1-2009, and B), parainfluenza virus 1–4, RSV, human metapneumovirus, and coronaviruses (229E, OC43, HKU1, NL63). Results for adenovirus, COVID-19 (SARS-CoV-2), and bacterial targets included in these assays were not evaluated in this study.

### Statistical Analysis

Patient and HA-RI characteristics were compared for the pre–universal masking period, universal masking period, and post–universal masking period. Categorical variables were reported as frequencies and compared using χ^2^, Fisher exact test, or univariate logistic regression, and continuous variables were reported using median (interquartile range) and compared using Kruskal-Wallis test, where appropriate. We compared patient demographics, HA-RI incidence, testing site, and the average monthly HA-RI rate per 1000 patient-days before, during, and after the implementation of universal masking (12-month, pre–universal masking period: 1 May 2019 to 30 April 2020; 13-month, universal masking period: 1 May 2020 to 31 May 2021; and 13-month, post–community mandate masking period: 1 June 2021 to 30 June 2022). Monthly composite HA-RI rates per 1000 patient-days were calculated and structural time-series model analysis with incidence rate ratios was performed to evaluate the role of intervention on the HA-RI rates during the study period, accounting for community-onset respiratory virus prevalence during the same period.

All statistical tests were 2-tailed, and significance was set at α = .05. Data were analyzed using SAS version 9.3 software (SAS Institute, Cary, North Carolina). This study was approved by the Washington University Human Research Protection Office.

## RESULTS

After exclusions, we identified 3015 hospitalized patients who had a positive respiratory viral test result either within 14 days prior to or during their stay. The median age was 58 years; 1448 (48.0%) patients were male, and 1724 (57.2%) were White. The median duration of hospital stay was 5 days. Four hundred and forty-one (14.6%) hospitalized patients had an HA-RI (Table 1). Among patients with an HA-RI, rhinovirus/enterovirus (51.0%) was the most common virus detected, followed by parainfluenza (14.3%), coronavirus 229E, OC43, HKU1, or NL63 (13.2%), and influenza A/B (10.0%) (Table 2). Most of the tests were performed on patients in the intensive care (31.1%), bone marrow transplant (28.8%), and medicine (23.4%) units compared to the outpatient setting, emergency department/observation, or admitting service among patients with a CO-RI (Table 2). Compared to CO-RI patients, HA-RI patients were slightly older, White, and more likely to be in the hospital for a longer period (*P* < .001; Supplementary Table 2).

**Table 1.** Demographic Characteristics of Patients Admitted to the Hospital With 1 or More Positive Respiratory Viruses Tested During Their Hospital Stay

Characteristic	Overall (n = 3015)^a^	Pre–Universal Masking (n = 1602)	Universal Masking (n = 391)	Post–Community Mask Mandate (n = 1022)	*P* Value
Age, y, median (IQR)	58 (37–67)	59 (41–68)	54 (35–64)	56 (35–67)	<.001
Sex					.006
Male	1448 (48.0)	807 (50.4)	190 (48.6)	451 (44.1)	
Female	1567 (52.0)	795 (49.6)	201 (51.4)	571 (55.9)	
Race					.036
White	1724 (57.2)	935 (58.4)	200 (51.2)	589 (57.6)	
Black	1173 (38.9)	601 (37.5)	180 (46.0)	392 (38.4)	
Other	118 (3.9)	66 (4.1)	11 (2.8)	41 (4.0)	
Length of hospital stay, d, median (IQR)	5 (3–11)	5 (3–11)	6 (4–12)	5 (3–10)	.045
First positive respiratory virus test					
Community-onset	2574 (85.4)	1368 (85.4)	319 (81.6)	887 (86.8)	.047
Mean monthly CO-RI rate per 1000 admissions	12.8	20.88	4.71	13.45	

Data are presented as No. (%) unless otherwise indicated.

Abbreviation: CO-RI, community-onset respiratory virus infection; IQR, interquartile range.

^a^Each admission was considered as a unit of observation.

**Table 2.** Healthcare-Associated Respiratory Virus Testing by Study Period

Characteristic	Overall	Pre–Universal Masking	Universal Masking^a^	Post–Community Mask Mandate	*P* Value
HA-RIs^b^	441/3015 (14.6)	234/1602 (14.6)	72/391 (18.4)	135/1413 (14.6)	.047
Mean monthly HA-RI rate per 1000 patient-days^c^	0.41	0.71	0.19	0.35	
HA-RIs by test order location					
Medicine	103 (23.4)	62 (26.5)	15 (20.8)	26 (19.3)	Reference
Surgery	29 (6.6)	16 (6.8)	4 (5.6)	9 (6.7)	.558
Neurology/neurosurgery	5 (1.1)	1 (0.40)	0 (0.0)	4 (3.0)	.028
Orthopedics	2 (0.5)	0 (0.0)	0 (0.0)	2 (1.5)	.990
ICU	137 (31.1)	73 (31.2)	28 (38.9)	36 (26.7)	.416
Cardiology	4 (0.9)	4 (1.7)	0 (0.0)	0 (0.0)	.988
BMT/oncology	127 (28.8)	64 (27.4)	18 (25.0)	45 (33.3)	.087
Gynecology/gynecological oncology	6 (1.4)	3 (1.3)	2 (2.8)	1 (0.7)	.862
Obstetrics	13 (3.0)	3 (1.3)	2 (2.8)	8 (5.9)	.007
Medical-surgical/observation/adult stepdown unit	11 (2.5)	6 (2.5)	3 (3.2)	2 (1.5)	.786
Psychiatry	4 (0.9)	2 (0.9)	0 (0.0)	2 (1.5)	.426
HA-RIs by organism detected of all positive					
Coronaviruses (229E, OC43, HKU1, NL63)	58/332 (17.5)	32/165 (19.4)	9/39 (23.1)	17/128 (13.3)	.243
Rhinovirus/enterovirus	225/1437 (15.7)	107/659 (16.2)	57/330 (17.3)	61/448 (13.6)	.327
Influenza A	41/392 (10.5)	34/270 (12.6)	0/0	7/122 (5.7)	.04
Influenza B	3/110 (2.7)	3/110 (2.7)	0/0	0/0	
Parainfluenza virus 1–4	63/314 (20.1)	25/132 (18.9)	5/11 (45.5)	33/171 (19.3)	.101
Metapneumovirus	21/161 (13.0)	13/104 (12.5)	0/0	8/57 (14.0)	.809
Respiratory syncytial virus	30/269 (11.2)	20/162 (12.4)	1/11 (9.1)	9/96 (35.7)	.746

Data are presented as No. (%) unless otherwise indicated.

Abbreviations: BMT, bone marrow transplant; HA-RI, healthcare-associated respiratory viral infection; ICU, intensive care unit.

^a^Universal masking implemented on 1 May 2020 (universal masking period: 1 May 2020 to 30 June 2022).

^b^HA-RI defined as unique first positive respiratory virus detected on day 3 or later of hospital admission.

^c^Using structural time-series model analysis.

### Pre–Universal Masking Period Versus Universal Masking Period Versus Post–Community Mask Mandate Period

**Figure 1.**
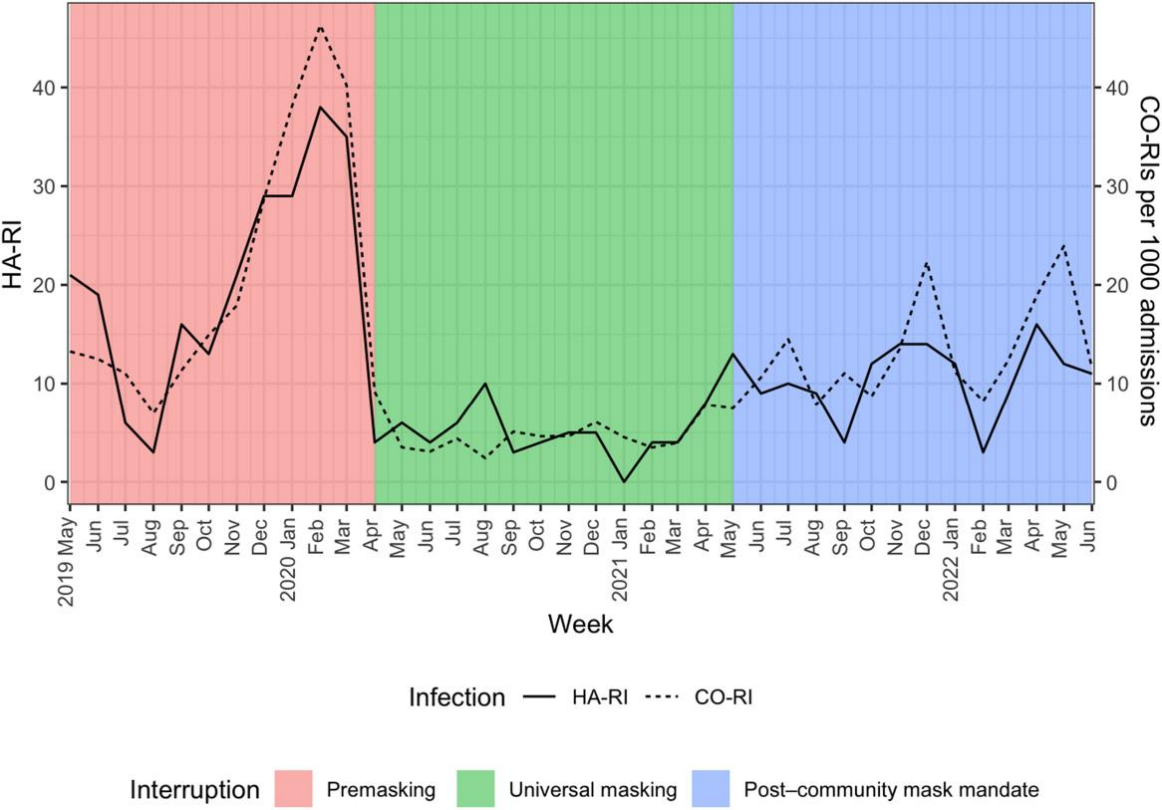
Community-onset and healthcare-associated respiratory virus testing per month for the study period. Abbreviations: CO-RI, community-onset respiratory viral infection; HA-RI, healthcare-associated respiratory viral infection.

Demographic characteristics for patients in the pre–universal masking period and the post–universal masking periods were similar, except a significantly higher proportion of females in the post–universal masking and post–community mandate periods (*P* < .001). We noted a significantly higher proportion of positive respiratory virus tests in the intensive care unit, the bone marrow transplant unit, orthopedics, and obstetrics and a lower proportion of positive tests in the medicine unit during the post–universal masking period compared with the premasking period. Of the 441 HA-RIs identified during the study period, 234 (53.1%) were during the pre–universal masking period, 72 (16.3%) were during the universal masking period, and 135 (30.6%) were in the post–community mask mandate period (Table 2). After controlling for monthly community-onset respiratory virus infection prevalence, the average monthly HA-RI rate significantly decreased after the implementation of universal masking (0.71 infections in the pre–universal masking period vs 0.27 infections per 1000 patient-days in the post–universal masking period; postintervention level difference, 5.77% and change in the slope, 0.33%; *P* < .001) (Figure 1).

## DISCUSSION

We observed a significant reduction in the average monthly HA-RI incidence per 100 patient-days during a hospital-wide universal masking period compared to the pre–universal masking period and adjusting for CO-RIs. While the intensive care unit and the bone marrow transplant service had a higher proportion of HA-RI cases in the post–community mask mandate period, significant reduction in the proportion of HA-RI cases was observed for the medicine units

Healthcare-associated respiratory virus infections are common and have a significant impact in the healthcare setting, resulting in increased morbidity, mortality, inappropriate antibiotic use, and costs [3]. These effects have been further exacerbated with the COVID-19 pandemic. Many institutions adopted aggressive infection prevention strategies such as universal masking, visitor restrictions, droplet and contact precautions, and other efforts to reduce HA-RIs, including COVID-19. Prior studies have shown that universal masking was effective in reducing HA-RIs, including COVID-19 [6, 11, 12]. At our institution, universal masking was implemented on 1 May 2020. All individuals (ie, patients, visitors, contractors, and hospital staff) aged >2 years were required to wear an isolation mask in all hospital areas, including clinical and common areas (eg, main hallways, waiting rooms). Masking compliance during the post–universal masking period was near 100% in all areas of the hospital for staff members, and visitors and patients were encouraged to mask in the rooms and during transportation. Findings from our study are consistent with prior studies evaluating the impact of universal masking on HA-RI rates [6, 11, 12]. A recent study by Seidelman and colleagues [13] evaluated the impact of hospital-specific infection prevention measures on the incidence of HA-RIs during the COVID-19 pandemic compared to prepandemic period and suggested that although the non–COVID-19 HA-RI rate decreased during the post–COVID-19 pandemic period, hospital interventions alone did not significantly reduce the incidence of HA-RIs. We noted a significant reduction in the average monthly HA-RI incidence per 100 patient-days during the post–universal masking period compared to the pre–universal masking period even after adjusting for CO-RIs. We also noted a slight increase in the HA-RI incidence rates in the post–community mask mandate period when compared to the universal masking period, and some of this transmission may be attributable to sick patients or visitors who were not 100% compliant with the hospital mask mandate. In a study of 4647 adults from Norway, wearing a surgical face mask in public places, over a 14-day period, was found to reduce the risk of the self-reported respiratory symptoms compared to those who did not wear a surgical mask [14]. Greenhalgh et al [15] performed a systematic review and meta-analysis of several primary studies and concluded that masks are effective in reducing the transmission of respiratory infections, if worn correctly and consistently.

Our study is limited using a retrospective study design and performance in a single academic hospital. Testing patterns observed in our institution may not be generalizable to other institutions. To minimize ascertainment bias from increased respiratory virus testing in the community at large during the COVID-19 pandemic, we estimated monthly, community respiratory viral prevalence based on patients who tested positive for infection prior to and within 3 days of hospital admission. We have obtained and analyzed hospital-level testing data for this study and have applied the National Healthcare Safety Network criteria for defining hospital-onset infections [16] using the 3-day cutoff. As a result, we may have misclassified some of the respiratory virus infections with shorter incubation period as community-acquired and those with a longer incubation period as hospital-onset infections. As we defined infection solely based on laboratory data, and we were unable to performed detailed chart review, we could not determine test indication or identify the respiratory symptoms that were present at admission, which may have led to misclassification as HA-RIs, due to delays in testing. As there were no COVID-19 infections during most of the pre–universal masking period, we were unable to draw conclusions on the impact of universal masking on COVID-19 healthcare-associated infections.

In conclusion, implementation of universal masking at our academic medical center during the COVID-19 pandemic was associated with a significantly reduced rate of HA-RIs, compared to usual practice. Implementation of universal masking during periods of increased community respiratory virus activity should be considered, along with other infection prevention measures, to limit the spread of HA-RIs within hospitals.

## Supplementary Data

Supplementary materials are available at *Open Forum Infectious Diseases* online. Consisting of data provided by the authors to benefit the reader, the posted materials are not copyedited and are the sole responsibility of the authors, so questions or comments should be addressed to the corresponding author.

## Supplementary Material

ofae617_Supplementary_Data

## Notes

***Patient consent*.** The design of the work has been approved by the Washington University Human Research Protection Office. This study does not include factors necessitating patient consent.

***Financial support*.** This research was supported in part by the Division of Infectious Diseases at the Washington University School of Medicine.
