# Supplementary material for: Effect of Universal Masking on Non–Severe Acute Respiratory Syndrome Coronavirus 2 Healthcare-Associated Respiratory Viral Infections
Source: Open Forum Infect Dis. 2024 Oct 14;11(10):ofae617. doi: 10.1093/ofid/ofae617 (PMC11521325; doi:10.1093/ofid/ofae617)
Supplement: ofae617_Supplementary_Data [file ofae617_supplementary_data.docx]

Supplementary Table 1. List of respiratory viral tests used at our hospital during the study period*

| **Test** |
| --- |
| Aries Flu A/B Assay (Luminex) |
| BioFire Upper Respiratory Panel 2.0 (Biomerieux) |
| BioFire Upper Respiratory Panel 2.1 (Biomerieux) |
| BioFire Pneumonia Panel (Biomerieux) |
| Roche SARS-CoV2 FluA/B Assay (Roche) |
| Xpert Flu/RSV Assay (Cepheid) |
| Xpert Flu/RSV/SARS-CoV2 Assay (Cepheid) |

*excludes testing platforms for SARS-CoV-2 only;

Supplementary Table 2. Demographic characteristics of patients with community-onset virus vs healthcare-associated respiratory virus

|  | Patients with Community-onset respiratory virus  N=2574 | Patients with healthcare-associated respiratory virus  N=441 | P-value |
| --- | --- | --- | --- |
| Age in years (median, interquartile range) | 57 (37-67) | 59 (43-68) | 0.02 |
| Sex |  |  | 0.522 |
| Male | 1230 (47.8) | 218 (49.4) |  |
| Female | 1344 (52.2) | 223 (50.6) |  |
| Race |  |  |  |
| White | 1430 (55.6) | 294 (66.7) | Reference |
| Black | 1041 (40.4) | 132 (29.9) | <0.001 |
| Other | 103 (4.0) | 15 (3.4) | 0.224 |
| Length of hospital stay in days (median, interquartile range) | 5 (3-8) | 17 (9-30) | <0.001 |
| Test order location |  |  |  |
| Medicine | 290 (11.3) | 103 (23.4) | Reference |
| Surgery | 144 (5.6) | 29 (6.6) | 0.015 |
| Neurology/ Neurosurgery | 9 (0.4) | 5 (1.1) | 0.432 |
| Orthopedics | 21 (0.8) | 2 (0.5) | 0.079 |
| ICUs | 342 (13.3) | 137 (31.1) | 0.431 |
| Cardiology | 9 (0.4) | 4 (0.9) | 0.714 |
| BMT/ Oncology | 296 (11.5) | 127 (28.8) | 0.226 |
| Gynecology/ Gynecological Oncology | 5 (0.2) | 6 (1.4) | 0.048 |
| Obstetrics | 212 (8.2) | 13 (3.0) | <0.001 |
| Med-Surgery/ ED/ Observation/ Admitting/ Adult step down | 808 (31.4) | 11(2.5) | <0.001 |
| Psychiatry | 2 (0.1) | 4 (0.9) | 0.048 |
| Outpatient setting | 436 (16.9) | 0 (0.0) | - |

*each admission was considered as a unit of observation
